# Treatment of Dental Caries, Complicated with Disorders of the Pulp and Peridental Membrane

**Published:** 1851-07

**Authors:** Robert Arthur


### AMERICAN JOURNAL

OF

### DENTAL SCIENCE.

**Vol. I. NEW SERIES—JULY, 1851. No. 4.**

### ARTICLE I.

*Treatment of Dental Caries, complicated with Disorders of the Pulp and Peridental Membrane.* By ROBERT ARTHUR, D.D.S.*

### NO. II.

### INFLAMMATION OF THE DENTINE.

THERE are few subjects connected with dental surgery, the study of which is of more practical importance than that exquisite sensibility of the dental bone, which so often presents itself even when caries of this part of the tooth is quite superficial. That this is a true inflammation of the part I am irresistibly led to conclude, both from a consideration of the structure of the teeth, and from the variety of phenomena which daily present themselves in practice, for which I am unable satisfactorily to account upon any other hypothesis.

I do not, at present, propose to enter very fully into the discussion of this question, but may on some future occasion do so. I will, however, state briefly the reasons which have brought me to the above conclusion.

The vitality of the bony portion of the teeth is not now questioned. It is, indeed, no longer a matter of theory, but has

* Entered, according to the Act of Congress, by ROBERT ARTHUR, D. D. S., in the Clerk's office of the District Court of Maryland.

been repeatedly demonstrated, and the presence of red blood in certain conditions of the teeth has been clearly displayed in the dentine. It has been shown, by the late Mr. Alexander Nasmyth, that the dentine is endowed with a much higher degree of vitality than is generally supposed. "The ivory," he says, "contains numerous fibres of animal matter composed of nuclei of cells arranged in a linear order, and these must be the agents of the transmission of sensation, although it must be admitted that no direct communication can be discovered between them and the nervous filaments; the fibres of the ivory must therefore be regarded as not only endowed with the functions of absorption, nutrition and secretion but also with that of sensation." That a tissue thus highly organized is liable to inflammation I do not think is to be questioned.

If it be admitted, and I think it cannot be successfully denied, that the dentine is liable to inflammation, then the question comes: under what circumstances is it likely to occur?

Among other exciting causes of inflammation enumerated in the chapter on that subject, introductory to "Cooper's Surgery," is mentioned "the application of innumerable irritating substances to parts." The agents in the mouth, which produce decay of the teeth, if sufficiently powerful to cause decomposition of the dental bone, which they do, cannot but be of an exceedingly irritating character. As the dental bone possesses all the vital qualities which render it liable to inflammation I cannot see why it should be expected to escape when exposed to the same influences which produce such an effect in other tissues of the body.

That this is really so, many phenomena of frequent occurrence, coming I suppose within the observation of every dentist in practice, clearly convince me.

This sensibility of the teeth is believed by many practicing dentists, at the present day, to be merely their natural sensibility.

If this were so, we might look for some uniformity in this condition of the teeth. We might at least expect to find the same degree of sensibility in all the teeth of the same individual; or if it be admitted that some of the teeth are endowed with a greater degree of vitality, which has been attributed to the incisor teeth, we might, at least, expect to find a higher degree of sensitiveness in these than in any others. But such is not the fact. And experience shows that in the mouth of the same individual, whilst some of the teeth are extremely sensitive, others, neither more nor less decayed, and of the same class of teeth, are scarcely at all sensitive.

If this extreme degree of sensibility of the teeth is truly their normal condition, we should expect to find in all cases the more nearly we approach the pulp cavity, an increase of sensibility. For no one will, I presume, dispute the principle that the natural sensibility of any part is in exact proportion to its degree of vitality; and we know that the nearer we approach the dental pulp the more vascular do we find the dentine. But we do not find the bone of a decayed cavity in all cases more sensitive as we approach the pulp, but often observe that the most sensitive part may be most distant from the pulp cavity; and it is a fact, which has been repeatedly stated, that if in some cases a layer of sensitive bone is cut rapidly away with a sharp instrument, the sound bone underneath will be found almost, if not entirely, insensible. This is exactly the reverse of what we should reasonably expect if the cause of this sensibility here mentioned were the true one.

It will sometimes be found that the cavity of a tooth very slightly decayed is so sensitive, that the lightest touch of an instrument, or a piece of cotton or lint brought into contact with it will produce the most violent pain. Whilst I know that the teeth, in a healthy state, are endowed with some sensibility, still I cannot believe that that which is here described is their natural condition.

It will be observed that when a cavity slightly decayed is prepared for filling, and the cavity is left open for twenty-four hours or longer, that the sensibility of the part is greatly increased; and I have noticed, too, that sometimes the bone of a cavity which is not at all sensitive, if left exposed, after having been prepared for filling, to the contact of the fluids of the mouth, it becomes, after a time, highly sensitive. I do not see how these facts can be accounted for on any other hypothesis than that which I have advanced.

At the time of writing, a well marked case of this kind has just occurred in my practice.

CASE I.—Mrs. C. aged about thirty-five. I had occasion to prepare for filling, amongst others, a cavity situated in the anterior lateral surface of the second left superior molar. The first molar was absent. The cavity was neither large nor very deep, and required considerable excavation; this produced no pain of consequence, not sufficient to induce the patient to speak of it. When the cavity was prepared, it so happened, that I was unable to complete the operation at that sitting. It was some days before I was able to see the lady again, and I then found the bone of the cavity so extremely tender, that it gave her great pain when I attempted to dry out the cavity, and I was obliged, or at least as it was in my power to accomplish it I preferred doing so, to remove this tenderness of the part before completing the operation.

On the other hand if a highly sensitive cavity is filled with any substance, so carefully as entirely to exclude the fluids of the mouth, this sensibility will after a time pass away, as is shown in the following case, which is one of by no means rare occurrence.

CASE II.—April 21, 1851. Miss H. aged about fourteen, came into my hands a year ago. Most of her teeth requiring attention were more or less sensitive, and she suffered much pain in the course of the performance of the necessary operations. The right superior lateral incisor was decayed, not very deeply, on the anterior lateral surface, but it was extremely sensitive, and with all her efforts, she could not bring herself to bear the perfect preparation of the cavity. After preparing it, however, as well as I could, I endeavored to fill it with gold, but found that the attempt caused so much pain that I desisted, satisfied that, if continued, the operation would result in the destruction of the vitality of the pulp. I then determined, to accomplish the object above indicated, to fill it temporarily, and tried gutta percha for the purpose. This, however, owing to the small size of the cavity, would not answer, although I am inclined to believe that in many cases it might be found useful. I then filled the cavity with tin foil, which with care, I succeeded in doing tolerably well. To-day, after the lapse of a year, she called on me again, as I had directed her to do. I removed the tin filling which was much firmer than I supposed it to be, and found that the sensibility of the bone, which was so extreme when I filled the cavity, had now almost entirely passed away. I had no difficulty in perfectly preparing the cavity for filling, although I cut down below the surface of the bone. I then filled it permanently with gold. There is another point about this case of importance. When the cavity was filled a year since, in the imperfect manner in which I was obliged to do it, the decomposed bone was not entirely removed, but a considerable portion of light colored caries (a species which, it is well known, proceeds with rapidity) was left, owing, as I have stated, to the extreme tenderness of the part; on removing the tin filling and examining the cavity, carefully, I could not detect the slightest change in its condition. If the caries had progressed at all, it had been so slowly, and to such an inconsiderable extent, that it was inappreciable. I shall subsequently refer to this fact.

Now, in this case what seems to have occurred; I not only cut away the external layer of bone which might have been supposed to be deprived of its vitality but I cut down to what must have been living bone, and found only a very slight degree of tenderness, and none of that exquisite sensibility which a year ago prevented me from operating well. I am satisfied that the bone, protected from the action of the extremely irritating agents which were rapidly destroying it, and producing great inflammation of the part, recovered its natural healthy condition.

I have intimated above that I feared, if I had persisted in filling the tooth in the case mentioned, that I should have run the risk of destroying the vitality of the pulp. And this result, sometimes following the operation upon a tooth in this condition, shows the propriety of considering this sensibility of the dentine in connection with the treatment of the dental pulp. I have found, as the following case will show, that the pulp is sometimes destroyed either by the pressure of a firm filling, well compacted, or the irritation attendant upon the preparation of the cavity. And I am inclined to believe that a good many of those cases we meet with so commonly, of teeth in which the pulp is dead whilst they are but slightly decayed and filled, are to be attributed to the same cause. I have often closely questioned persons who have had teeth in this condition, and have been satisfied from what I have gathered from them that, at the time the teeth were filled, the pulp could not have been even slightly exposed. My own experience confirms me in this view. I have generally been very cautious in all such cases. Sometimes treating them as the one above detailed, and sometimes taking measures first to remove this excessive sensibility, and consequently few such results as that recorded in the case below have occurred in my practice. I do not mean to be understood as implying that consequences of this kind are likely to occur in a majority of cases where this sensibility exists, but I am satisfied that they do occur probably more frequently than is generally suspected.

CASE III.—Mrs. L. Aged about thirty-five. First left superior bicuspis-crown and posterior lateral surface. This tooth I found slightly decayed at the places above indicated, but so extremely sensitive that it required the greatest effort on the part of the patient to bear the necessary excavation. As, however, she was willing to bear the pain attendant upon the operation, I completed the preparation of the two cavities. Fearing, from the extreme tenderness of the cavities, that the pulp might be slightly exposed, I now most carefully examined them in every part, and satisfied myself that this was not so. I then proceeded to fill them with gold, a good deal of pain was experienced during the performance of this part of the operation, but not more than I have many times observed in similar circumstances. After the fillings were completed, no pain was complained of, and the patient went away quite satisfied to have got through with so painful an operation. After the lapse of some days she called again, and complained of having suffered some pain with this tooth. I examined it, and to my surprise found that an alveolar abscess had formed above it. I intended removing the fillings and extirpating the pulp, but I did not have an opportunity of doing so.

In this case I know perfectly well that the pulp was not exposed, even very slightly. The caries was by no means deep, not extending I am quite sure half way through the dentine, and if it had not been for the very great tenderness of the cavity, I should never have thought it necessary to examine to see if the nerve was exposed. But supposing it possible I did make a most careful examination before beginning to fill, and quite satisfied myself that there was no opening into the pulp cavity. This it seems to me was a clear case of inflammation of the bone, extending to the pulp, and the unfavorable result was caused by the great irritation consequent upon the excavation of the cavity and the pressure of the gold filling, which, of course, was inserted with much force, upon the inflamed bone.

Careful attention to the symptoms attendant upon the progress of caries of the dentine, furnishes, to my mind, strong evidence of the position I have here taken. I have had the good or bad fortune to be able to study this subject in my own particular case, and whilst my sound teeth are as insensible as those of any other person, those which have begun to decay, furnish me, by sensation, with unmistakeable evidence that they are diseased. I am aware, at times, of a sensation in them, not exactly a painful one, but a faint consciousness of the form and position of the decayed cavity. My attention has frequently been called by patients to teeth which they had an impression were decayed, although they had never caused pain nor exhibited externally any evidences of decay. I never neglect on receiving such a hint, most carefully to examine the teeth indicated, and rarely, if ever, find them sound and healthy. This impression of a sensation is produced, I do not doubt, by the compression of the inflamed and swollen animal portion of the bony tissue, in a manner precisely analogous to what occurs in tooth-ache in the pulp itself. Dr. Maynard informs me that he has been conscious of the same sensation under similar circumstances.

In the lecture on dental caries in the admirable treatise on "Dental Physiology and Surgery," by Mr. Tomes, republished in the "Journal," I find, since writing the above, the following paragraph corroborative of the statement just made.

"The diseased action is not attended by severe pain, but I am disposed to think that even early in the disease, there is, in almost all cases, a feeling of slight uneasiness in the affected tooth, though the degree may be so slight as to escape detection at the time, or lead only to the supposition that some foreign matter has got between the teeth. I am induced to adopt this opinion, though at variance with several authors of distinction, both from the description given by patients who have suffered from caries, and also from personal experience in this unpleasant malady. On several occasions my attention has been drawn to a tooth by a sensation not perhaps amounting to pain, but to slight discomfort only, which lasted but a few minutes. After a recurrence, from time to time of this unpleasant sensation, I have examined the tooth, and have invariably found that caries had commenced in the situation from whence the uneasiness seemed to proceed." Mr. Tomes' experience seems to have been precisely my own.

There is a great difference in different individuals with respect to the sensibility of teeth affected by caries. Whilst many suffer the most agonizing pain in having operations performed upon their teeth, many suffer so slightly as to render it a matter of indifference, and others do not experience the slightest painful sensation. I have noticed that in cases where the caries is of soft consistence and light color, denoting rapid decomposition of the part affected, great tenderness of the bone is present, and on the contrary, when the caries is of a dark color, and progresses slowly, that there is but little tenderness of the affected part. This, however, is not invariably the case, but I have observed it generally. The explanation of this difference must, I think, be found in the fact, that in one case the chemical decomposition of the bone, produced by agents which do not in the same proportion destroy the animal portion, goes on so rapidly that the death of the vital part does not take place simultaneously, but is previously thrown into a state of great inflammation; in the other case, where the disease progresses slowly, the entire death of the whole bone occurs.

I have observed that in the teeth of the same individual there is a difference in degree of this sensibility at different periods of life. I have now in my hands, a lady about twenty-eight years of age, upon whose teeth I have operated since she was a girl. Before her marriage, her teeth were generally as little sensitive as those of any other person. At this time she has been married several years, and has had two children. Her teeth, when decayed, are now extremely sensitive.

It is a well known fact, that in some cases, if the bone for a little distance below the decayed surface of a very sensitive cavity be cut away with a sharp instrument, it will be found but slightly tender, and the operation may be continued with but little pain to the patient. Now, it seems to me, that no clearer evidence of a change in the condition of the bone could be asked for than this; for if this sensitiveness were only the natural sensibility of the tooth, it would of course not grow less as you approach the more vital parts of the tooth, but would with reason be expected to increase. What we understand by inflammation, is such a change in the condition of the part as will fully account for the fact here mentioned. The irritating agents causing decay, have excited an inflammatory action in the portions of bone exposed to its contact, without affecting such portions as are beyond its reach, and as soon as this outer layer is removed, we reach bone which only exhibits its natural degree of sensibility.

It is not always the case, however, that the removal of the carious portion and the layer of bone immediately adjacent to it will have the effect just mentioned; but this extreme sensitiveness may continue till the operation is completed. This may be accounted for on the supposition that the contact of the carious bone has excited so high a degree of inflammation that the whole body of the dentine is affected.

Other facts going to confirm the opinion that this great sensitiveness of the carious dental bone is due to inflammation of the part, might be mentioned, but this I do not think necessary. If those already presented are not as convincing to others as they have been to me, I should be glad to have them explained, for I must confess that I have never been able, nor am I yet so after thinking a great deal about it, to find any other satisfactory explanation than that which I have just given.*

It may be said, that the discussion of this question is a matter of very little practical importance. I do not think so. If my position is tenable, it may, as I have stated, account for instances in which the destruction of the pulp has occurred, after an operation, long before it has become uncovered, and it must lead to other practical considerations of importance. Besides, it is often seen that the discovery of truth, in relation to the causes of any morbid condition of the system, leads to practical results which were never before anticipated. This remark, indeed, holds good with regard to the proper and thorough scientific examination of every subject. A discussion of this kind, based upon observation, is a very different thing from the practice, not uncommon, of erecting an elaborate theoretical structure, which cannot be applied to any useful purpose, upon a very slender foundation of facts.

But whatever may be the real cause of the condition of the teeth here alluded to, and about which I have said more than I intended, it is one of the most serious obstacles to good practice with which we meet; at least I find it so. And I know that some skilful members of the profession, with whom I have conversed on the subject, have found it equally so. It may be a great weakness to allow the knowledge that you are inflicting severe pain in performing an operation to disturb your equanimity, and in any way interfere with the perfect accomplishment of what you know is intended for the ultimate good of your patient; but it is a weakness I have found it very difficult to overcome. Although I make it a rule, in this as in every other case, to do my best, regarding, as little as possible,

* Since writing the above, I have read an article in the "American Journal of Dental Science," by Dr. E. Townsend, of Philadelphia, in which I am gratified to find, he takes the same view which I have just presented.

the pain inflicted, if it is necessary, I must confess, that I am so anxious to get through with so unpleasant a task, that I am apt to stop in the preparation of a cavity, which I *think* it will answer, without always being certain of it. Gentlemen who have alluded to this subject, have generally treated it lightly, making some passing remarks about it, and stating that a few bold strokes, with a sharp instrument, will end the difficulty. In some cases, as I have already said, this is so; but not, by any means, in the greater number. I may, perhaps, give more importance to this matter from having known something of it from personal experience. My own teeth are very sensitive, and I do assert, for those who have never had the benefit of such experience, (to those who have had, I am sure no such assurance is necessary,) that the preparation of a decayed cavity for filling, when the bone is inflamed, is one of the most agonisingly painful operations that I have ever undergone. It is a peculiar, indescribable, unendurable sort of pain, very different from that produced by the incision of the skin or of muscular tissue. These cases are far from being exceptions. In the course of practice I frequently meet with persons who seemed to suffer the most dreadful agony from this cause. That this experience is not peculiar to my own practice, the following extract will show: "A few cases are met with, in which there is considerable pain attending the progress of caries long before the pulp is at all affected by exposure, or by the contact of partially decomposed dentine. The tooth is, from the commencement, highly sensitive: the contact of hot or cold fluids induces severe pain, and may attempt to remove the carious dentine, though the disease has existed but for a short time, and is very slight in extent and depth, *produces such unbearable pain, that the attempt is for the time obliged to be abandoned.*"—Tomes, Jour. vol. viii, 211. In many of these instances, this exhibition of suffering could not be attributed to a lack of firmness, as the following case, I think, will prove:

CASE IV.—Some six years ago, my services were desired by a lady whose teeth were in such condition that they were, with the exception of the inferior canine and incisor teeth, past the hope of preservation. These were slightly decayed on their lateral surfaces. The patient in question had never had any teeth extracted, and at three sittings they were all removed, with the exception of those just mentioned. It was determined that these should be filled. She bore the operation of the extraction of her teeth, some of which were very difficult to take out, with stoical firmness. After the mouth had healed somewhat, I proceeded to prepare the cavities in the remaining teeth for filling, and found them extremely sensitive. She shrunk at once from this pain, and after I had completed one cavity and had begun another, she declared she *could not* bear it, and insisted on having the teeth extracted. As their preservation was not a matter of much importance, and as I then knew of no safe means of removing this sensitiveness, I complied with her request. This is a strong case; but I have repeatedly met with patients who have declared that if it were not for the loss of the teeth, they would infinitely prefer to suffer the pain of their extraction, than to endure this horrible torture.

Now, the operation of the extraction of teeth, even with the present greatly improved instruments in use for the purpose, is an exceedingly painful one, and probably as much dreaded by most persons as any kind of surgical operation to which they could be obliged to submit. And, I think, it will be admitted that the operation which exceeds this in the intensity and duration of the pain it causes, even if it be only in the imagination of the patient, is well worthy of our most earnest efforts to find a remedy for the evil. It is a plain duty, and it ought to be a source of great pleasure to every one, to do all in his power towards the alleviation of human suffering; and there is no field in which he may labor more profitably for this purpose than in this one. For when the vast number of operations on the teeth, which we daily perform, are considered, it seems probable that the aggregate of pain inflicted, is greater than occurs in all the practice of general surgery. This, indeed, would be certain if all persons who are obliged to submit to operations on the teeth found them in the condition just described, for whilst a large proportion of the people composing our communities now give attention to their teeth, a comparatively small number are subjected to surgical operations.

But, however regardless a practicing dentist may be of the pain he inflicts, or however steadily and coolly he can go on to the completion of his operations, when he is aware that his patient is in an agony of suffering, there are other considerations which render an effort toward the lessening of this evil, an important matter to him. Although some persons who come into his hands have nerve enough to bear any operation upon their teeth, no matter how painful it may be, without flinching, there are others who cannot bear it steadily, and some who cannot, or will not, bear it at all. And it is easy to perceive, that any thing like a perfect operation is out of the question, when the patient is shrinking from, and writhing under, every touch of an instrument.

The very great dread of dental operations exhibited by children, is to be attributed principally to this cause; wholly, indeed, with the exception of the extraction of teeth; and I have met with children who could not, by any means, be induced to submit to the operation of filling their sensitive teeth. I remember one case, in which the little fellow who made a great effort to bear the pain, sobbed so violently at every attempt I made to prepare a cavity in one of his teeth, that I was obliged to desist. And at this time, the case of a little girl, not more than seven years of age, for whom I found it necessary to fill the first permanent molar teeth, comes into my mind. She sat perfectly still whilst I was operating, but the big tears rolled down her cheeks during the whole time, giving such touching evidence of what she was suffering, and of the firmness with which she bore it, that it was no little relief to me to have completed what I felt obliged to do for her.

Now it will be seen, that if a certain and safe remedy for this be found, it will not only render our profession less formidable, but will enable us to perform more perfect operations. It must be plainly seen, too, that it will greatly increase the aggregate amount of business, and contribute largely towards the cause of humanity by extending the sphere of our usefulness; for many persons who cannot now be induced to avail themselves of the benefits of our profession, in consequence of their invincible dread of the pain they are obliged to endure, would gladly do so if they could escape some portion of it.

This matter has engaged my earnest attention for a number of years, ever since, indeed, I have been in practice, and I have not failed to make trial of every thing which has been suggested by others, or which has occurred to my own mind, for the purpose indicated; but until within a few months past without satisfactory success.

I have tried faithfully, the mineral acids, the caustic alkali, nitrate of silver, tannic acid, chloride of zinc and even the actual cautery.

Chloride of zinc, which I first used about seven years ago, and applied for experiment in a variety of ways, did not at all come up to my expectations. In the first case in which I used it, the result was so strikingly successful that I was satisfied that the desideratum for which I had been looking, had at last been fallen upon. But I subsequently found that, from causes which I could not discover, it was so extremely uncertain in its effects, not answering the desired purpose once in twenty times, and gave so much pain when applied, that I abandoned its use. I gave it a faithful trial, and was reluctant to give it up, for I felt convinced, from its peculiar escharotic properties, that it was just the agent which would answer fully the purpose of destroying the vitality of the part with which it was placed in contact, without extending any further. I perceive it stated in the last number of the Journal, by Dr. E. Townsend, of Philadelphia, that he uses it with success, by allowing it to remain half an hour in the sensitive cavity. I certainly still believe it well worthy of further trial.

Till within a few months, the only substance of which I knew any thing which would, with uniform certainty, remove this sensitiveness of the dental bone, was arsenic. But there are serious objections to this agent for the purpose in question, and it could scarcely, with safety, be recommended for general use. When applied to a tooth considerably decayed, or allowed to remain any considerable length of time in a *very* superficial cavity, it is likely to pass in a state of solution through the substance of the bone, to the pulp, and ultimately destroy the vitality of this part of the tooth. This result, so liable to follow its application, has driven arsenic, for this purpose, out of general use. It has been asserted that arsenic cannot be applied to any tooth, in any stage of decay, without eventually causing the destruction of its vitality, even after the lapse of years. This is supposed to occur from the impossibility after it is once applied of removing every particle of the substance from the cavity, and that, however minute the quantity which remains, it will at last find its way to the pulp. But this assertion I know, from actual experience, to be like many other sweeping assertions occasionally made, as well in our own as in other professions, to be unfounded in fact. I have used arsenic occasionally for the last ten years for this purpose, and have never in any case, when I applied it with proper precaution, and my directions were carefully observed, had bad results. Cases I know now of seven years standing where the teeth are as healthy as any in the mouth. But it is unnecessary to urge the claims of this substance, for the purpose in question, or to give any particular directions about its use, for I have another agent to offer which entirely supersedes the necessity of using it in any case.

This is the ore of cobalt. My attention was first called to this mineral, for the purpose of destroying the vitality of the dental pulp, by Dr. Hunter, of Cincinnati, in a conversation with him last summer in Philadelphia. Dr. H. did not claim it as a discovery of his own, but said he had accidentally fallen upon it, and that it had been used in the west long before he knew any thing of it. I have since used it exclusively for destroying the vitality of the pulp, and latterly with very satisfactory results, for removing that sensibility of the bone which has formed the subject of this chapter.

The active escharotic properties of the ore of cobalt is undoubtedly due to the arsenic which is always found combined with it in large proportions. Some kinds of this ore contain as much as fifty-five per cent. of pure arsenious acid. But the peculiar advantage of this substance is, that it is insoluble in the fluids of the mouth, whilst the arsenious acid is readily soluble. The importance of this difference between the two substances, is obvious. The danger of using arsenic in the cases of which we are now treating, arises from the fact, that it is soluble in the fluids of the mouth and, in a state of solution, as we have already said, will rapidly pass through the substance of the bony portion of a tooth, when placed in a cavity, and reach the pulp; this it will certainly do, if allowed to remain long enough, no matter how thick or dense the layer of bone may be which covers the pulp. It has been said, and I doubt not with truth, that if an opening is made through the enamel of a sound tooth and a small quantity of dry arsenic is placed in it, that it will eventually destroy the pulp. Now, although I am unable at present to demonstrate that the particles of pulverised cobalt cannot pass through the substance of the dental bone, I think it was doing no violence to strong probability to suppose that it could not, and felt justified in making experiments with it. The animal matter of the bony substance is small, and does not, under ordinary circumstances, admit the corpuscles of red blood, which have (see Carpenter's Physiology, p. 120) the minimum diameter of 1.2800th of an inch. It is scarcely probable, as I have said, that the particles of cobalt in a pulverized state can be so minute as to pass through a substance which excludes the smallest corpuscles of the red blood.

The inferences drawn from actual experiment in the use of cobalt for the purpose indicated, and a comparison with the effects produced by the arsenious acid in similar circumstances, justify strongly such a conclusion.

The following is one of several similar cases which have come into my hands:

Case V.—January 10th, 1851. Mrs. A. aged twenty-seven; nervous temperament. Found first left inferior bicuspis badly decayed on the anterior lateral surface. Examined closely and found decayed very nearly to the pulp, although this was not actually uncovered at any point. On attempting to prepare it for filling, found the bone of the cavity extremely sensitive, and the patient, who did not lack firmness, declared after several efforts, that it was entirely out of her power to bear the pain necessary to prepare it for filling. She begged me to extract the tooth at once and let the matter be over. I found I could do nothing, unless by some means this sensibility could be removed, and I regarded it as quite justifiable to attempt it, even at the risk of destroying the pulp. I had used cobalt for destroying the vitality of the pulp, had thought a good deal about its action in such a case as this, and determined to make a trial of it. I mixed a small quantity finely pulverized, with gutta percha, softened by the lamp, and filled the cavity with it. This I allowed to remain twelve hours. At the end of this time I removed it, and found the sensibility of the cavity scarcely at all diminished. Confident that it must destroy the vitality of the bone with which it was placed in contact, I again applied it, and allowed it to remain twenty-four hours. On its removal, I found the bone still sensitive, but remembering from some experiments with arsenic, for the purpose, that the sensibility of the bone is always at first exalted by the action of the material, but subsides in some hours after its removal, I determined to wait a little while before making another trial. About forty-eight hours were now allowed to elapse before I made another examination, when I found so little of the sensibility remaining that I was enabled to complete the operation with scarcely any inconvenience to the patient.

Now, if arsenious acid had been used in this case, I know well, from the experience I have had in its use, that in one hour it would have reached the pulp. The symptoms produced would first have been a dull sensation of pain, and if the arsenic had not been removed at once, would have increased in violence till tooth-ache, of the worst character, would have set in. If it had not produced any immediate effect of this kind, it would certainly have occurred in a few days.

That the cobalt, in this case, did not produce any effect upon the pulp is evident, from the fact that no kind of uneasiness followed its application, although it remained in all, in contact with but a very thin layer of bone between it and the pulp, some thirty-six hours. But if this is not sufficient, the subsequent condition of the tooth fully proves, I think, the fact, that its vitality is unimpaired. Some four months have now elapsed since the operation was completed, and there has not yet occurred the slightest symptom indicating any change in its condition. I have examined it carefully, and repeatedly, and have not yet seen the least indication of peridental inflammation or anything which would induce me, for a moment, to believe that the pulp had been touched.

This, as I have stated, is but one case out of several of precisely the same character.

The cobalt ore will, undoubtedly, uniformly remove this extreme sensibility of the dental bone; and the only question as to the propriety of its introduction into general use in practice is: can it always be used with safety, without lessening the chances of the ultimate preservation of the teeth? This is a question which I cannot yet venture, with confidence, to answer affirmatively. In the course of my experience I have met with many things which at first sight promised a great deal, and which were eventually shown to have objections almost, if not entirely, neutralizing the good results expected from them. This may be the case with the agent I now offer. The advantages which in my hands have attended its use, almost seem to be too great not to have some drawback. But I am satisfied, that even if it do not fully meet my expectations now, it will be found a valuable acquisition to the practice of dental surgery. I can only say to every member of the profession disposed to make a trial with it, to do so cautiously and with discrimination, and time, alone, must test its real value.

The only unpleasant result I have yet observed in its use, so far, is, that in some cases slight peridental inflammation has occurred. What renders this unaccountable, is, that I have always observed it to occur, when it did occur, which has probably not been more than in six cases out of a hundred, in quite superficial cavities, and never, that I now recollect, in any case of considerable decay, where it might have been expected. It is quite impossible to account for this result by supposing that the cobalt could have, by any means, passed through the thicker and denser body of bone in these cases, when it did not do so when placed in a cavity where there was a much thinner wall of bone to protect the pulp, and this, too, of a character (the fibres being more direct and not so tortuous, according to Nasmyth) much more likely to favor the transmission of any substance which could pass through it than the bone nearer the enamel.

I have applied it in several ways, and have observed some difference in the results corresponding to the manner of its application. I found it by no means so active when mixed with wax, as I once used it, the wax coming probably between the cobalt and bone, and preventing direct contact.

The most convenient and effective method I have fallen upon to apply it, is to dissolve sandarach in alcohol, saturate a piece of cotton in the solution, and take upon it as much cobalt as is necessary; about the twentieth part of a grain is sufficient. Enough cotton should be taken to fill the cavity. When pressed into the cavity, the superabundant liquid must be wiped away, and enough will be left to cement the fibres of the cotton and form a very substantial temporary filling. This will make it easy to apply the cobalt to the most shallow cavities, as the cotton saturated with this preparation will readily stick to the surface to which it is applied.

When the cavity is so deep as to give occasion for any fear of its action upon the pulp, I have always regarded it as safer to apply it with gutta percha, by softening this substance and working the cobalt into it. Even if it be possible for the particles of cobalt to pass through the substance of the bone, this would effectually prevent it; for the gutta percha is perfectly insoluble in the mouth, and whilst it would hold the cobalt in contact with the affected part, would not allow a single atom to escape from its grasp. It is used, as any one will know, who has used gutta percha for any purpose, by softening the gum in a lamp and pressing it into the cavity. Gutta percha dissolved in chloroform, might answer a better purpose than the sandarach mentioned above; I have not tried it, however.

In using the cobalt for this purpose, I generally allow it to remain from twenty-four to forty-eight hours. If the cavity is deep, I remove it in twenty-four hours, and fill in twenty-four hours afterwards. I have generally found, in removing it, after it had remained only this long, that the sensibility of the part was not much diminished, but would be found entirely gone, if the same time were allowed to elapse after its removal before the preparation of the cavity was attempted. I have observed, as I have already mentioned, in the use of arsenic, for the same purpose, that in removing it, after it had remained two or three hours, the sensibility of the part would be found rather exalted than diminished; but, that the next day scarcely a trace of it would be found remaining. In bringing about the death of the bone, the inflammation of the part to which it is applied, is, of course, at first increased. But if the cobalt is allowed to remain forty-eight hours, no trace of the sensibility complained of will generally be found to remain. Whether it is always safe to do this, must be a matter for future observation; I can scarcely think any risk attends it, for in slight cavities, on one or two occasions, I have, in consequence of failure of the patient to come at the time appointed, allowed to remain a week without observing any bad consequences.

[To be continued.]

